# Manure as a Potential Hotspot for Antibiotic Resistance Dissemination by Horizontal Gene Transfer Events

**DOI:** 10.3390/vetsci7030110

**Published:** 2020-08-13

**Authors:** Tiago Lima, Sara Domingues, Gabriela Jorge Da Silva

**Affiliations:** 1 Faculty of Pharmacy of University of Coimbra, University of Coimbra, 3000-458 Coimbra, Portugal; tiagoventurall@gmail.com (T.L.); gjsilva@ci.uc.pt (G.J.D.S.); 2 Center for Neuroscience and Cell Biology, University of Coimbra, 3004-517 Coimbra, Portugal

**Keywords:** manure, soil fertilization, horizontal gene transfer, antimicrobial resistance, mobile genetic elements, One Health

## Abstract

The increasing demand for animal-derived foods has led to intensive and large-scale livestock production with the consequent formation of large amounts of manure. Livestock manure is widely used in agricultural practices as soil fertilizer worldwide. However, several antibiotic residues, antibiotic resistance genes (ARGs) and antibiotic-resistant bacteria are frequently detected in manure and manure-amended soils. This review explores the role of manure in the persistence and dissemination of ARGs in the environment, analyzes the procedures used to decrease antimicrobial resistance in manure and the potential impact of manure application in public health. We highlight that manure shows unique features as a hotspot for antimicrobial gene dissemination by horizontal transfer events: richness in nutrients, a high abundance and diversity of bacteria populations and antibiotic residues that may exert a selective pressure on bacteria and trigger gene mobilization; reduction methodologies are able to reduce the concentrations of some, but not all, antimicrobials and microorganisms. Conjugation events are often seen in the manure environment, even after composting. Antibiotic resistance is considered a growing threat to human, animal and environmental health. Therefore, it is crucial to reduce the amount of antimicrobials and the load of antimicrobial resistant bacteria that end up in soil.

## 1. Introduction

Livestock production is an extremely dynamic activity, highly influenced not only by human population growth, but also by competition for natural resources and by human health and environmental concerns. In fact, the human population in 2050, according to the Department of Economic and Social Affairs of the United Nations, is estimated to be 9.735 billion [1], with the consequent increasing demand for livestock products [2]. The need to increase the potential of livestock production has led to developments in breeding, nutrition and animal health [3]. Therefore, antibiotics are not only used globally with prophylactic and metaphylactic purposes and for the treatment of animal infectious disease, but are also used in subtherapeutic doses to promote animal growth [4,5].

The administration of antibiotics to animals through feed and drinking water, or by other routes such as injection, has become increasingly important in intensive food-animal production [4,6,7]. Despite restrictions on the use of antibiotics in animal feed as growth promoters, some of these compounds are still used illegally on some small farms [6]. In European Union countries, the use of antibiotics as growth promoters was banned in 2006 [8].

The extensive use and misuse of antimicrobial agents in human and veterinary medicine led to the emergence and selection of antimicrobial resistance in bacteria, with potential adverse consequences for human and animal health [9]. Resistant bacteria and their genes can disseminate between humans and animals by the food chain and spread in the environment, which is the reason why antimicrobial resistance must be handled by a holistic approach, a concept designated as One Health [10]. After administration, many antibiotics that are used in food-producing animals are poorly absorbed in the animal gut, resulting in their excretion into the environment, without degradation, in their active metabolite forms [6].

The increasing demand for animal-derived products has led to intensive and large-scale livestock breeding, with the consequent production of a huge amount of livestock manure [11]. Animal manure is directly used as organic fertilizer in the agricultural sector and consists, mostly, of animal feces [12]. This became a common practice in many countries of the world as an alternative to chemical fertilizers for arable soils of low fertility [13,14].

Due to anthropogenic activities, livestock manure may act as a reservoir for antibiotic residues and bacteria carrying different antibiotic resistance genes (ARGs) that confer resistance to many clinically important antibiotics [14,15]. The main focus of the current review will be to explore how manure can work as a hotspot for antimicrobial resistance gene dissemination. Manure gives to the soil a unique environment for the spread of antibiotic resistance genes by horizontal gene transfer (HGT) mechanisms [16,17]. It is rich in nutrients and presents dense and highly diverse bacterial populations and accumulates antibiotic residues and/or metals that act as a selective pressure and may trigger the exchange of bacterial DNA. These elements mix with agricultural soil bacterial communities and irrigation water, reaching microbial communities from different ecosystems, including water streams, and spread to the environment, wildlife and human and other animal communities [18]. Herein, we will discuss the composition of manure, highlighting the most frequent antibiotics and ARGs associated with mobile genetic elements and the HGT mechanisms described so far in manure, to better understand how manure can be a source of antimicrobial resistance spread, the potential impact in public health and the procedures used to reduce this risk and their limitations.

## 2. Composition of Manure

The intensive production of meat, milk, eggs and other agricultural products for increasing human consumption needs results in the generation of large amounts of manure during animal growth. The mixture of animal feces, urine, feed remains, soil, wash waters and bedding materials to absorb waste such as wood chips, wheat straw, flax straw, sawdust or even peanut and rice hulls, give rise to the product that has been used as organic soil fertilizer on several farms [11]. Manure from different animals have different physical properties, different nutrient contents and specific application rates on the land. This complexity of manure contents makes it more difficult to manage when compared to chemical fertilizers [19].

In addition to the nutrients excreted by animals, including nitrogen, phosphorus, potassium and sulfur, manure also contains heavy metals, such as cadmium, cobalt, copper (Cu), lead, manganese, nickel, selenium and zinc (Zn), which are considered long-term soil contaminants since they are not degraded [19,20,21,22].

Furthermore, the heavy metals present in manure may exert a long-term and continuous selective pressure on ARGs via co-resistance or cross-resistance because the resistance genes for antibiotics and metals are often located together in the same plasmid or other mobile genetic element (MGE) [23]. A strong relationship between MGEs, metals and metal resistance genes and ARGs present in manure has been previously determined [24]. Therefore, heavy metals may also promote the long-term persistence of ARGs during manure management, storage and disposal [25,26,27].

The use of manure as a fertilizer not only provides nutrients and organic material during agricultural practices but also facilitates the spread of beneficial or pathogenic microorganisms in soils [28,29]. In fact, the bacterial communities of manure and soil are highly diverse [30]. The microbial population present in manure is highly diverse [29], and mainly depends on the animal species [31]. Manure is an important source of bacteria, which can cause serious illness both in animals and humans. The major manure-borne pathogens are zoonotic bacteria, such as *Salmonella* spp., *Escherichia coli*, *Campylobacter* spp., *Listeria monocytogenes*, *Yersinia enterocolitica*; and protozoa like *Cryptosporidium parvum* and *Giardia lamblia* [32]. Other manure-borne pathogens and phages are also present, but they are more unusual [33]. Moreover, *Enterococcus*, *Bacillus* and *Clostridium* species are also found in manure [29]. These bacteria are colonizers of the intestinal tract of animals and are shed with feces. Their potential dissemination into environment and humans firstly depends on the ability to survive in manure after excretion, which can vary from a few days to several months. For instance, *Bacillus* spp., due to the ability to form spores, can resist unfavorable growth conditions for long periods of time [29]. Moreover, several *Salmonella enterica* serotypes were shown to persist in soil for at least 21 days after manure application, while they were rarely present before [34]. The application of manure also leads to the spread of these bacteria in the environment, namely in water [18].

Animal feces and manure are important reservoirs of antibiotics and antibiotic-resistant bacteria. Nowadays, ARGs are considered as emerging contaminants, considering the potential risks and their origin, mobility and elimination in the environment [35]. The use of antibiotics during animal growth favors the appearance of antibiotic-resistant bacteria in the gut microbiota, which consequently may be released within feces and be disseminated by manure application [30]. The application of organic fertilizers, as well as inorganic fertilizers to a less extent, has been shown to impact the soil microbial community and it is the major driver of the shaping of the antibiotic resistome [36,37].

### Antibiotics and Antimicrobial Resistance Genes in Manure

A strong correlation between the high use of certain antibiotics in livestock (and their residues) and the presence of ARGs and MGEs, such as plasmids, integrative conjugative elements, transposons and integrons, involved in the spread of ARGs via HGT pathways from manure to soil microbes, has been found [38,39,40]. ARGs and MGEs, especially integrons and transposons, in manure have been showed to be closely related, and their increased abundance is related to manure application [24,37]. Manure has a higher impact on the abundance and diversity of ARGs in soil than chemical fertilizers [37].

As most antibiotics have polar functional groups and high solubility in water, many antibiotics used in livestock production are poorly absorbed in the animal gut, resulting in the excretion of 30–90% of the parent compound via feces or urine [5]. Antibiotic metabolites can also be excreted with antimicrobial activity or can return to the initial active compound [41]. One example is the conjugation in the liver of sulfamethazine with sugars after its administration; after its excretion, bacteria are able to degrade the sugars and convert it back to its bioactive form [42]. Therefore, active forms of veterinary antimicrobials are often found in manure and may disseminate in the environment after its application on soil [5]. Yet, the stability and efficacy of antibiotics in the environment depends on their physio-chemical properties, types of soil, climatic conditions and other environmental factors [5,43]. Non-degraded antibiotics in manure and in soil may act as a selective pressure and contribute to the emergence and dissemination of antimicrobial resistance determinants [44].

In general, ARG concentrations in livestock waste is higher than in human waste, and in manured soil, levels of ARGs can be 28,000 times higher compared to un-manured soil [35]. However, antibiotic-resistant bacteria are also abundant in manure from animals with no history of antibiotic treatment, suggesting that the animal gastrointestinal microbiota harbor intrinsically antibiotic-resistant bacteria [14].

One hundred and nine ARGs, associated with resistance to antibiotics extensively used in livestock farming and representing the main classes applied in human and veterinary medicine, have been detected in fresh chicken, pig and bovine manure collected from 12 large-scale Chinese farms [24,45]. ARG abundance and content may differ depending on the species [24,35,45,46]. For instance, in the previously mentioned Chinese farms, a higher diversity and abundance of ARGs was seen in chicken manure, followed by pig manure and finally by bovine manure [24,47], while dairy manure collected from two Canadian farms had a slightly higher variety of ARGs [46]. Common to both studies is the observation that the species is not the only influencing factor of ARG distribution, as this varied from farm to farm and with the sampling year. Dairy manure slurry also had higher levels of ARGs than a mix of dry stack equine, bovine and ovine manure [48]. In addition to the antibiotic administration patterns [49], feed administration, exposure to heavy metals, animal age, time and manure nitrogen content may all contribute to the ARG content in manure [24,46,48]. The major concern about the presence of ARGs in manure is the possibility of their transfer to the soil bacteria via HGT mechanisms, which can promote the spread of antimicrobial resistance among different microbial communities [30,37,48]. In this context, it is important to understand the differences in the content of antibiotic-resistant bacteria and ARGs in soils in response to manure application. This response mainly depends on the characteristics of applied manures [50]. For instance, swine manure application was shown to have a higher impact on the soil ARG frequency than dairy manure [46]. Different ARG compositions were found between pig and chicken feces. For example, sulfonamide-resistant *Escherichia coli* from different host species had significantly different distributions of the resistance determinant *sul2* gene, which was found less frequently in swine samples than in other animals’ samples [51]. After a one-time manure application, persistence times in soil vary among different ARGs [48]. However, ARGs are stable in the microbial community of a soil that regularly receives manure applications [36,37,52].

Diverse classes of antibiotics are used in food-animal production, which depends on the animal, the objective of the given antibiotic (therapeutic, prophylaxis, growth promotion) and country policy rules.

Tetracyclines and sulfonamides are two classes of antibiotics often used in veterinary medicine due to their broad-spectrum activity, relatively low toxicity and low price [53,54]. After administration, most tetracyclines are excreted as active compounds through feces and urine, which are spread in the environment by manure fertilization practices [55,56]. These compounds have a high affinity for soil organic matter [57]. Additionally, animals’ gut microbiota show a very high prevalence of tetracycline resistance [30,54].

The *tet* gene family, involved in the active efflux of tetracycline compounds, ribosomal protection or the enzymatic modification of antimicrobial agents [58], has been reported as one of the most frequently detected ARGs in animal manure [21,25]. After manure application, *tet* gene levels in the soil are increased significantly and manure application amended with tetracyclines promotes the accumulation of *tet* genes and modifies the soil bacterial composition [54,55,59,60]. These genes are often embedded in plasmids (isolated from manure and animal bacteria) [61,62] and inserted in transposons, which facilitates their dissemination [54]. For example, the genes *tetW*, *tetO* and *tetQ* are very common in pig and cattle manure, suggesting that they are stably maintained in the animals’ gut microbiota [63].

Sulfonamides have been widely used in clinical and veterinary medicine to treat bacterial and protozoal infections [64]. They inhibit the dihydropteroate synthase (DHPS), inhibiting the folic acid biosynthesis pathway, which is necessary for the synthesis of DNA and RNA precursors, leading to the non-replication of bacterial DNA and the non-synthesis of bacterial proteins [65]. To date, three *sul* genes have been identified [66]: *sul1* is frequently identified in class 1 integrons in slurry and soil environments, *sul2* was first identified on a broad host range *Escherichia coli* plasmid and has been found on small nonconjugative resistance plasmids of the IncQ family, while the *sul3* gene has been identified in isolates from different sources that also carry a class 1 integron [64,65,67]. Nowadays, *sul* genes occur in a wide range of bacterial species, because they are often located on transposable elements of self-transferable or mobilizable broad host range plasmids [68,69].

In contrast to tetracyclines, sulfonamide compounds do not sorb strongly to the soil, and have been detected in surface water, groundwater and soil pore water [70]. Nevertheless, these antibiotics and their resistance genes have been found in soil and manure [21,52,64,68,71,72]. Mutations in the chromosomal DHPS gene that codes for the inhibition of folic acid biosynthesis (*folP*) or the acquisition of an alternative DHPS gene (*sul*) are responsible for sulfonamide resistance [64,65]. The *sul* genes occur in a wide range of bacterial species because they are frequently located on mobilizable plasmids and in mobile genetic elements, such as class 1 integrons and transposons [64,65,67,68,69]. These elements carry multiple antibiotic resistance genes that are co-selected by sulphonamides [64,69,72]. The presence of *sul* genes in pig farms and cattle waste lagoons seems to be associated with the use of sulfonamide [30,73]. Sulfadiazine was found in turkey and chicken manure in significant amounts [74], suggesting that poultry manure could be a source of *sul* genes. Several studies reported sulfonamide resistance genes as the most detected ARGs in manured soils. In general, the *sul1* gene is the most frequently detected ARG in manures, manured soil samples, agricultural and non-agricultural soils, wastewater and surface water in feedlots, evidencing their extensive ability to spread [75,76].

β-lactams are one of the most widely used groups of antibiotics, including in veterinary medicine [77]. β-lactam residues, β-lactam-resistant bacteria and β-lactam-resistant determinants have been found in dairy cattle manure because β-lactam antibiotics are often used to treat mastitis [14,15,40,78,79]. Pig manure can also work as a reservoir for the transferable amoxicillin antibiotic resistance genes *bla*_TEM_. These are often embedded in IncN plasmids [40]. IncN plasmids are also associated with *bla*_CTX-M_ genes, coding for extended-spectrum β-lactamase (ESBL)—which have been found in bacteria from pigs, farmers and farm environments, such as manure [80]. These findings may suggest that IncN plasmids play a key role in β-lactamase gene dissemination in manure and soil bacteria. Beyond their insertion in mobilizable plasmids, ESBL genes are located in insertion sequences, like IS*Ecp1* and IS*CR1*, which facilitate their spread [81].

Macrolide antibiotics, such as erythromycin and tylosin, are often administered together with lincosamides and streptogramins in livestock production [82,83]. Tylosin is not completely metabolized in the gut and up to three quarters of the antibiotic can be excreted in the urine and feces [84]; tylosin residues were reported in swine manure [83,84]. Macrolide-resistant bacteria, carrying erythromycin ribosome methylation (*erm*) genes and/or macrolide efflux (*mef*) genes, are also excreted in feces [83,84]. Moreover, the binding site for erythromycin overlaps binding sites for other macrolides, lincosamides and streptograminB (MLSB) antibiotics, leading to cross-resistance in the MLSB antibiotic family due to *emr* encoded genes [83]. Therefore, the use of tylosin increases the resistance to MLSB of animal gut microbiota [85]. Specifically, its use at sub-therapeutic concentrations has been correlated with increasing *Enterococcus* spp. macrolide resistance [86]. Several studies report *erm* genes in manured soil [82,87,88,89,90]. Swine manure seems to have a higher content of erm genes than bovine manure samples [85], and a high diversity. Various *erm* genes have been found in swine waste lagoons: *ermA*, *ermB*, *ermC*, *ermF*, *ermG*, *ermT*, *ermQ* and *ermX,* with *ermB* and *ermF* being the most prevalent. Remarkably, the *ermB* gene was never detected in non-manured soils or before the first spring manure amendment, which is strongly suggestive of the introduction of this gene into soil via manure application [89]. The *erm(B)* gene has often been found to be associated with the presence of *tetM*, probably because these genes are frequently integrated together on the Tn*916*-Tn*1545* family of conjugative transposons [91].

Colistin is an old antibiotic widely used in veterinary medicine for the prevention and treatment of gastrointestinal infections caused by *Enterobacteriaceae* [92,93]. Colistin is poorly absorbed through the pig gastrointestinal tract and is subsequently excreted in feces. It not only contaminates the soil but may also contribute to the development and spread of colistin resistance by exerting pressure selection on animal gut microbiota [94]. Recently, the plasmid-mediated and mobilizable colistin resistance genes, *mcr*, have been identified from bacteria worldwide [92,93]. Ten different *mcr* genes (*mcr-1* to *mcr-10*) and variants have been described [95], even though the *mcr-1* gene is the most reported worldwide, especially in animal samples. This may suggest that the colistin mobilizable resistance gene reached humans via *mcr* from animal bacteria [96]. The presence of colistin in manure correlates with the finding of the *mcr-1* gene both in pig and poultry manure samples [96,97]. Additionally, this gene has been detected in bovine and horse manure [98]. Livestock manure seems to be an important reservoir of *mcr*-encoding plasmids, mostly carried by colistin-resistant *Escherichia coli* on diverse plasmid replicon types: IncX4, IncI1, IncFII, IncFIB, IncX1 and IncQ1 [97,99,100].

Quinolones are also used in intensive animal production and might be found in manure. The most frequent resistance is encoded by mutations in the topoisomerase genes, involved in DNA replication, which are chromosomal. Yet, there are plasmid resistances genes, such as in the *qnr* family, that may confer resistance to quinolones when mutations in the chromosome are already present [101]. They can be transferred to a recipient cell, but they might not give clinical resistance to the host cell.

## 3. ARGs Spread by HGT Mechanisms

HGT is the process of the transfer of genetic information between organisms that are not directly related, and includes the spread of antibiotic resistance genes among bacteria [102]. There are three main mechanisms of HGT in bacteria: conjugation, natural transformation and transduction, which involve plasmids, naked DNA and bacteriophages, respectively. Moreover, MGEs, such as integrons, insertion sequences and transposons, also play a synergistic role in resistance dissemination [16]. Conjugation is considered the most common mechanism for HGT between different bacteria and occurs when a donor bacterium directly transfer genes to another recipient cell, while in transduction and natural transformation, there is no direct contact between the donor and recipient cells [103].

HGT can promote rather large-scale changes in a bacterial genome, allowing for the evolution of genes and bacteria, which in the ultimate instance may result in the emergence of resistant bacteria [104].

Fertilized soils may be hotspots for HGT occurrence once all necessary elements are present, including ARGs provided by manure, the natural soil resistome, bacterial cells vehiculated by manure that may also carry ARGs, soil microbiota and antibiotic residues from manure and irrigation water that create a selective pressure in bacteria. The presence of antibiotic residues, even at subinhibitory concentrations, can stimulate HGT events and ARG transfer [105]. Dong et al. showed that extracellular ARGs, such as those detected in manure, maintain their transforming ability [106]. Animal manure represents a major source of antibiotics and ARGs in the environment [107], creating a favorable environment for HGT events [37,48]. Organic fertilization has been demonstrated to accelerate ARG dissemination in soil by HGT events, as compared to inorganic fertilization [36]. Furthermore, manure is often stored before being applied to soil, allowing for HGT and resistance selection during storage. The application of a mathematical model concluded that it is crucial to know HGT rates, antibiotic influx and the length of storage time in order to prevent the spread of resistance; for instance, reducing antibiotic inflow only controls resistance if the gene transfer rate is low [108].

The co-occurrence of heavy metals and antibiotics in manure may increase the HGT pathways of ARGs and promote an increased emergence of antibiotic-resistant bacteria [23]. Many studies show not only a positive correlation between the presence of many ARGs and Cu levels in soil, but also the ability of Cu to promote the transfer of ARGs via conjugation [109]. The antimicrobial properties of Cu may explain its ability to promote conjugative transfer not only within bacterial genera but also across different bacterial genera. The overproduction of reactive oxygen species, elevated SOS response and enhanced cell membrane permeability might be the underlying mechanisms in the increase of conjugative activity [103]. In particular, *sul1* and *sul2* genes are strictly associated with levels of Cu, Zn and mercury. Other specific ARGs, such as *tetM*, *bla*_CTX-M_ and *bla*_OXA_ genes, are more closely associated with chromium; *tetW* with nickel; and *tetM* with nickel, iron and lead levels [21,110]. Finally, quaternary ammonium compounds used as common disinfectants in pig farms may also enhance the co-selection of ARGs [111].

The rapid evolution, proliferation and spread of antibiotic resistance is especially promoted by plasmid mobilization via conjugation [112]. Conjugation requires physical cell-to-cell contact, with the donor being responsible for the formation of the sexual (F) pilus. Due to this contact, the bacterial environment and cell density influence the outcome frequency of conjugation events [105]. In general, plasmids are composed of the core genes required for essential functions, such as replication, transfer and maintenance, and accessory functions, encoding antibiotic or heavy metal resistance, catabolic functions and virulence or pathogenicity determinants; they often have a mosaic structure [40]. There are several worldwide reports of plasmids harboring extended-spectrum beta-lactamase (ESBL) genes, *ampC* genes, quinolone resistance genes (*qnr*), *mcr* genes and carbapenemase genes from environmental samples, including manure [112]. These plasmid-borne genes are mainly located on broad host range IncP-1, IncQ, IncN, IncW and IncF replicon-type plasmids, which are important vectors for the dissemination of ARGs between distantly related genera and species [30,105,112,113]. *Salmonella* plasmids were shown to persist for long periods in a North Carolina commercial swine farm environment after land manure application; 14 strains belonging to six serotypes were able to transfer ARGs embedded in plasmids to *Escherichia coli* JM109 by conjugation, and sequencing analysis revealed that plasmid-mediated ARG transfer occurred among the different *Salmonella* serotypes [112]. This result highlights that conjugal transfer occurs in the field. Plasmid pSN1216-29 has been isolated from cow manure [114] and was demonstrated to be able to conjugate with a broad range of bacterial genera extracted from soil and cow manure [115]; despite the fact that this plasmid does not carry accessory genes, one transposition event can lead to antimicrobial resistance acquisition, followed by horizontal dissemination.

The *traF* gene, involved in the assembly of the F pilus in *Escherichia coli*, has been detected at a high rate in multidrug-resistant *Escherichia* spp. from livestock manure, suggesting the possible occurrence of high levels of conjugation with the consequent spread of ARGs [23].

As mentioned above, the use of wastewater and manure for the irrigation and fertilization of soil may be the main uses responsible for the vast dissemination of the *mcr-1* gene. Recently, two *Escherichia coli* harboring *mcr-1* were isolated from horse (*n* = 1) and bovine (*n* = 1) manure, and the plasmid carrying *mcr-1* isolated from the horse manure was successfully transferred by conjugation [116]. The *sul1* and *sul2* genes from pig slurry and manured agricultural soil samples were transferred from a variety of Gram-negative bacterial species to *Escherichia coli* K-12 CV601 and *Pseudomonas putida* UWC1 recipient cells by conjugation at different rates, indicating their presence on different mobile elements [64]. Livestock manure has a high prevalence of class 1 integrons carrying different ARGs as gene cassettes. The association between integrons and the *sul1* gene may contribute to the dissemination of sulfonamide resistance in arable soils [117]. The presence of an antibiotic and/or its residues in manure increases the activity of integrases and transposases and consequently increases the excision/integration of gene cassettes in integrons [30].

A wide dissemination of *bla*_CTX-M-1_ has been reported in *Escherichia coli* in pig farming, including animals, manure and farmworkers. Despite the high diversity of the isolates, *bla*_CTX-M-1_ was associated with similar conjugative and broad host range IncN plasmids, suggesting their horizontal mobilization between the different isolates [80].

Plasmids belonging to different incompatibility types and carrying a variety of resistance genes, including *bla*_TEM-1_, *sul1*, *sul2* and *sul3*, have been isolated from piggery manure, with most of them being able to transfer by conjugation, highlighting the potential for the transfer of resistance genes in manure [40].

Lateral *erm* gene transfer in a manure environment has also been reported between Gram-negative and Gram-positive bacteria. The molecular analysis of *ermB* genes from human, swine and poultry *Enterococcus faecium* isolates suggests that the HGT of antibiotic resistance genes plays a greater role in the dissemination of resistant determinants than the direct transmission of resistant strains [118]. The soil-borne equine and zoonotic pathogen *Rhodococcus equi* has been shown to be able to conjugate with other environmental Actinobacteria that share the habitat, leading to macrolide resistance due to the dissemination of *erm*(46) embedded in plasmid pRErm46; conjugation between two *Rhodococcus equi* strains also occurred in equine manure at environmental temperatures (22 and 30 °C) [119].

LowGC type plasmids are a group of conjugative plasmids that are abundant in pig manure and manured soils [62,120]. The accessory regions, which encode ARGs and mobile element-derived genes, have highly variable composition. However, after conjugative transfer, this plasmid structure is genetically unstable, which may have contributed to the significant diversity of this type of plasmid [62,121]. LowGC plasmids are closely involved with the horizontal dissemination of *sul* genes in manure and soil and have a long-term persistence in manured soils [62,120]. These plasmids can spread to a wide range of bacterial species, and *sul2* gene can integrate into the chromosome through an intracellular mechanism mediated by insertion sequences located in the plasmid [122]. Jechalke et al. demonstrated that the effect of swine manure containing an antibiotic, in this particular case sulfadiazine, on the horizontal dissemination of *sul* genes embedded into LowGC plasmids also depends on the soil traits (bulk soil or rhizosphere) and the developmental stage of the plant species [120]. LowGC plasmids may also be involved in the dissemination into the environment and soil of *tetY* genes [121]. *Acinetobacter* species were identified as natural hosts for LowGC plasmids in manure and soil. This finding is particularly relevant since *Acinetobacter* spp. comprise both environmental and nosocomial species and the spread of ARGs located on LowGC plasmids is facilitated between closely related bacteria [111,121].

The conjugative Tn*916*-like transposon, associated with tetracycline resistance, has been identified in two isolates belonging to the *Bacillus cereus* group isolated from pig manure, and transfer to *Enterococcus faecalis* JH2-2 by conjugation was demonstrated [123].

The transfer of plasmid-mediated tetracycline and gentamicin resistance from indigenous soil bacteria to *Pseudomonas putida* KT2442 by conjugation has been shown to occur immediately after the amendment of soil with cattle manure, while the transfer rate after 29 weeks was insignificant when compared with unfertilized soil; the application of other fertilizers, such as sewage sludge and municipal solid waste compost also had similar effects [17]. This result highlights that the duration of persistence of ARGs in soil is not crucial, and the increase in ARG abundance due to manure application, even if for a short period of time, is enough to promote the horizontal dissemination of antimicrobial resistance.

A conjugative plasmid carrying a multidrug efflux pump encoding gene, *oqxAB*, especially associated with olaquindox and previously extensively used as a growth promoter in pigs, and chloramphenicol resistance have been detected in *Escherichia coli* isolated from swine manure [124]. This plasmid has since been shown to be transferred to *Escherichia coli* N43 by conjugation [125].

Transduction is the process of the transfer of genetic material between bacteria through the action of a virus, named a bacteriophage or simply a phage. Phages have been determined to be the most abundant biological entities on Earth and are a driving force in bacterial diversity and evolution [126], since they are able to regulate the dynamic of the bacterial population as well as facilitate HGT events. It is estimated that phage-like elements have been involved in the acquisition of 20% of the bacterial genome [127]. Agricultural soils are rich in bacteria and phages, in a ratio 1:10 to 1:100 [128], and manure slurry has been shown to have a higher prevalence of phages than other environmental sources [129]. Environmental phages harbor ARGs and genes conferring resistance to aminoglycosides, β-lactams and sulfonamides have been detected in fertilized soils [130]. There are studies that demonstrate the transmission of virulence genes by transduction in soil [18] or of resistance genes through a phage isolated from sewage effluent [131]; however, there are no studies exploring the involvement of transduction in the transfer of resistance genes within agricultural soil, despite the high potential for the occurrence of this HGT mechanism in this environment [18].

Natural transformation is characterized by the acquisition of naked DNA from the environment by competent recipient cells. Extracellular DNA has been shown to maintain its transformability over long periods of time [132], and there are several naturally competent bacterial species that inhabit soil, such as *Acinetobacter baylyi*, *Bacillus subtilis* and *Pseudomonas stutzeri*, or are part of the animal digestive tract, like *Campylobacter* spp. [133]. Several studies demonstrated that DNA adsorbed to different particles of the soil is protected against nuclease activity, which allows it to persist unchanged and available for uptake by competent cells [105,133]. Calcium is essential to the natural transformation of some species, and its presence in some natural environments has been shown to induce natural competence. Swine manure has a high calcium content, which suggests that natural transformation can occur in this environment [105], though there are as yet no studies demonstrating this.

A fourth HGT mechanism, transformation mediated by membrane vesicles (MVs), has been proposed [134]. MVs are released from all living cells, are biologically active and contain different components, such as genetic material, including chromosomal and plasmid DNA, as well as different types of RNA. These vesicles can mediate the transfer of ARGs between bacteria and can persist over time in natural environments [134,135]. Environmental and external stimuli induce the release of MVs; for instance, the exposure to subinhibitory antibiotics of different classes may induce MV release and HGT mediated by MVs [134]. Although there are no studies focused on the presence of MVs in manure and their potential involvement in HGT events, manure and manured soils can be a privileged platform for HGT mediated by MVs due to the presence of antimicrobials in manure and the stress response triggered by the land application of manure in soil resident bacterial communities [135].

The acquisition of antibiotic resistance genes and MGEs through HGT pathways often has a biological cost for the host cell in terms of reduced growth, competition and/or infectivity [136,137]. This metabolic burden often reduces the competitiveness of host bacteria in the absence of antibiotic pressure [30]. However this fitness reduction can be ameliorated within a few generations after plasmid acquisition through the evolution of compensatory mechanisms, which may restore fitness without compromising resistance to antibiotics [30,136,137]. Due to the long-term persistence of antibiotic pressure, organic farming practices may have favored the maintenance of acquired genes by HGT pathways in host bacteria of soil and/or manure and, consequently, this may have led to antibiotic-resistant bacteria emergence and persistence in the manure environment [30].

## 4. Manure Treatment

The control and elimination of antimicrobial resistance in agricultural fields are urgently needed [138]. The sewage treatment, composting or anaerobic digestion process of manure are some of the techniques that are used to reduce the concentrations of antimicrobials, ARGs and microorganisms that are introduced in soil through manure fertilization practices [139].

Composting is a spontaneous and biological process of aerobic digestion that involves the mineralization and humification of organic matter [12,140]. This bio-oxidative process involves environmental microorganisms, which break down organic materials, resulting in the formation of a stable final product, known as compost [12,140,141]. Composting is a suitable option for manure management, which implies the reduction of volume of the animal wastes and the elimination or reduction of pathogenic microorganisms, with economic, environmental and public health advantages. The composting procedure requires controlled conditions. Parameters such as bulk density, porosity, particle size, nutrient content, carbon/nitrogen ratio, temperature, pH, moisture and oxygen supply determine the optimal conditions of microbial development and organic matter degradation [12].

The elimination of antibiotic residues seems to be effective during this process [12]. For example, chlortetracycline and sulfadiazine residues were completely removed from antibiotic-spiked swine manure within 21 and 3 days, respectively, while 17–31% of spiked ciprofloxacin remained in the composting mass. Similar results were observed by Esperón et al. in antibiotic-spiked poultry manure, with a 90% decrease in the concentration of ciprofloxacin and doxycycline after composting for 3 weeks [141]. In addition, β-lactams have a very sensitive ring structure which is easily cleaved by the phosphate, ammonia and hydroxyl ions present in the composting environment, resulting in a degradation product with no antimicrobial activity [142]. Unfortunately, the elimination of ARGs may not be as straightforward.

The abundance of ARGs in livestock manure during composting is highly variable, essentially due to three main factors: the reproduction or death of intestinal microorganisms carrying ARGs in animal manure; the content of antibiotic residues and heavy metals that may exert selective pressure on ARG-carrying bacteria; and, finally, the possibility of the horizontal spread of ARGs [143]. Several studies have investigated changes in ARG and MGE content during composting, but the conclusions are inconsistent. For instance, the abundance of *tetC, tetG, tetW, tetX, sul2, drfA1, drfA7, ermB, ermF, ermQ*, *ermX* and *intI1* genes after chicken manure composting with the addition of bamboo charcoal for 26 days was found to decrease significantly, while *sul1* increased [144]; the composting of poultry manure for 10 weeks also led to a significant reduction of *tetA, tetB tetK, tetM, tetQ, tetS, tetW, ermB, qnrS* and *bla*_TEM_ genes and an increased abundance of *sul1*, *sul2*, *tetY* and *aadA* genes [141]. The absolute abundances of *intI1* and *intI2,* genes coding for integron integrases, and different *erm*, *sul* and *tet* ARGs were reduced by up to 45% after the composting of pig manure with cotton stalks, with good aeration, for 40 days [143]. Thermophilic composting was shown to be an effective method for the elimination of the *mcr-1* gene in livestock manure, which was completely undetectable after 22 days of composting at a high temperature (44–65 °C) [98]. Furthermore, *bla*_TEM,_ *sul3* and *ermB* gene abundance decreased significantly during the composting of chicken manure in comparison to simple storage for 6 weeks [145]. In contrast, the abundance of *tetG, ermF, and tetA* did not change during composting, while there was an increase in *tetW* and *tetO* [146]. Different MGEs were also detected in most of the bovine, chicken and pig manure composts [24].

Obviously, composting conditions play a key role in the success of ARG elimination in manure. Temperature is pointed out as a critical factor for ARG reduction during composting because high temperatures may kill most of the bacterial species [47]. However, if thermophilic microorganisms are the hosts of ARGs, their abundance may increase during the thermophilic stage of composting [143].

The inconsistent results obtained by these studies call for the improvement of this process by adjusting the composting parameters and by adding adsorbents/surfactants or decomposition agents. The addition of natural zeolite during composting may be able to reduce some ARGs and accelerate the removal of pathogenic bacteria in compost, minimizing environmental risks [147,148]. This effect could be due to the porous structure and ability to reduce selective pressure and ARG co-selection by heavy metals, which implies decreased rates of microbiological contact and then HGT through the reduction of conjugation [147]. Similar effects are observed with the addition of biochar during chicken and swine manure composting due to its ability to increase the temperature, thereby prolonging the thermophilic phase. Moreover, biochar decreased copper and zinc levels with a consequent reduction of co-selection pressure by heavy metals [144,149]. However, the efficiency and the extension of the removal of ARGs is dependent on the type of biochar and manure [149]. Superabsorbent polymers have also been shown to be useful in the reduction of ARGs and MGEs in swine manure compost [150]. The application of an iron-based material and phosphate-dissolving inoculant during composting appears to be a promising method for the efficient removal of ARGs. On the other hand, the addition of red mud hindered the removal of tetracycline resistance genes and affected the shaping of bacterial communities during composting [151].

Anaerobic digestion is another widely used technique for manure treatment, associated with the reduction of organic matter pollution, microbial pathogens and veterinary antibiotic residues and the production of methane-rich biogas, a renewable energy source [152]. However, there are no consistent results and no key conclusions concerning the fate of ARGs during the anaerobic digestion process [153]. Some studies have pointed to anaerobic digestion as an alternative to reduce ARG content in different animal manures, although their complete removal has rarely been achieved [152,154,155,156,157]. The efficient removal of ARGs seems to be temperature dependent, since in thermophilic conditions (55 °C) ARG removal rates are higher than in mesophilic conditions (35 °C) [152]. For instance, *tetM*, *tetQ*, *gyrA* and *sul1* levels were reduced with high efficiency during the anaerobic digestion of dairy manure with a high temperature treatment, while the increase in Bacteroidetes was responsible for the increases in *tetC*, *tetM*, *tetQ*, *tetX*, and *sul1* under moderate and mesophilic temperatures. Moreover, the inhibition of HGT at high temperatures may also contribute to the decreased content of ARGs in the thermophilic system [155]. In another study, the relative abundances of *intI1, sul1, sul2, tetA, tetO and tetX* were evaluated during thermophilic and mesophilic swine manure anaerobic digestion conditions. All these ARGs decreased in abundance at the thermophilic temperature, while only *intI1, sul1* and *tetO* decreased and *sul2*, *tetA* and *tetX* increased at the mesophilic temperature [157].

Furthermore, it is generally considered that anaerobic digestion could reduce the content of the *ermB* gene in swine manure, but in sewage sludge, the *ermB* gene generally increased after anaerobic digestion [153]. The substrate matrix types may also influence the efficiency of the ARG removal process due to the different physiochemical proprieties of different animal manures [153,158]. On the other hand, the microbial community needed for the degradation of different animal manures may be distinct and its evolution is, at the same time, conditioned by the presence of ARGs and the selective pressure of antibiotic residues [153].

In general, reduction techniques appear to be a sustainable and environmentally friendly process to reduce the risk of antimicrobial resistance dissemination and its impact on environmental and public health. Yet, conjugation was shown to occur even after 6 weeks of chicken manure composting [145], suggesting that reduction techniques are still not completely able to abolish the horizontal transfer of ARGs, although they can reduce it [159]. Therefore, more studies on composting parameters are needed to improve its efficiency.

## 5. Impact on Human, Animal and Environmental Health/One Health Perspective

Antimicrobial resistance is recognized as one of the “One Health” challenges that requires close observation and implementation strategies along the potential human–animal–environment bacterial spread chain [139,160].

**Figure 1:**
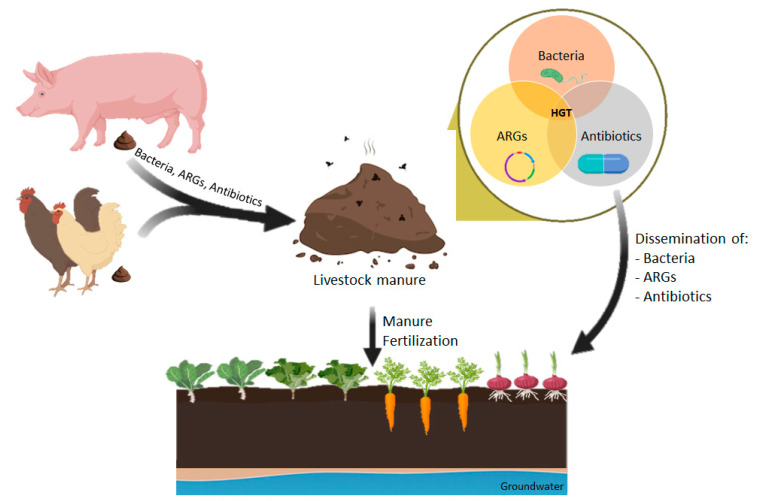
Dissemination of antimicrobial resistance associated with manure application in agricultural soils.

The introduction of manure containing antibiotics and ARGs into the environment has a significant effect on the spread of resistance in the human community because human microbiota and pathogens may acquire MGEs carrying antibiotic resistance determinants by HGT events, even between distantly related species [30,111]. The environment acts as a bridge to different compartments; animal to manure to soil to water to sediment and, at the same time, it acts as a reservoir of MGEs that may interact and spread to other compartments or to human and animal hosts (Figure 1) [161].

Resistance determinants can be transferred from animal to animal or animal to human either directly via contact, or indirectly through the food chain, water, sludge-fertilized soils and manure. The human transmission of antibiotic-resistant bacteria and ARGs from agricultural sources is mostly foodborne [139]. Antibiotic-resistant bacteria are often found in vegetables and fruits which are cultivated in animal-manured soil [162,163]. Since these fresh products contaminated by manure or irrigation water are often consumed raw, their intake may result in the ingestion of resistant bacteria which may colonize the human gut or pass through the intestine and pose a threat to public health [163,164]. The microbial communities of fresh produce reflect the soil where it grows. Two studies reported a higher frequency of β-lactam-resistant bacteria in manure-amended soil and raise the possibility that these β-lactamase encoded genes could spread from manure-amended soil bacteria to human pathogens [14,79]. Food-producing animals, and their related food products in all stages of processing, also contain abundant quantities of antibiotics, resistant bacteria and their resistance genes that spread into humans through food chain, increasing the possibility of resistant determinant exchange between human, environmental and animal microbiomes [165]. Furthermore, in countries with poor water treatment and sewage conditions, there is an increased risk of manure-borne resistant bacteria and resistant gene transmission from animals [139].

The widespread use of antibiotics in human clinical settings and the agricultural and livestock industries has exerted a selective pressure for the selection and survival of antibiotic-resistant bacteria [166]. The survival and spread of antibiotic-resistant bacteria and ARGs is a threat and a major concern for human, animal and environmental health due to the disastrous impact on clinical infection outcomes [139,166]. Antimicrobial resistance is one of the major causes responsible for the reduction of antimicrobial therapy effectiveness and the increasing incidence, severity and costs of infections in clinical settings [139].

Moreover, anthropogenic activities have a great impact on the environmental resistome because large amounts of antimicrobials are produced annually and, through diverse pathways, such as manure fertilization, are spread into the environment (Figure 1) [139]. Thus, intensive animal production is often considered the major cause responsible for the increased environmental burden of antibiotics [167,168]. Antibiotics in the soil can also cause adverse effects in soil plants [35,167]. It is crucial to reduce the amounts of antimicrobials that end up in soil, either due to the prudent use of antimicrobials or by composting techniques.

Overall, manure must be seen has a hotspot for the dissemination of antibiotics, metals and antimicrobial resistance genes, with impacts on public and environmental health, and measures must be taken to reduce their impact on society.

Preventive actions to decrease the risk of antimicrobial resistance should keep being implemented [160]. In the livestock sector, the maintenance of good animal health should be encouraged by implementing practices such as decreased animal density in feedlots and improved nutritional programs [35].

Finally, the development and implementation of Good Agricultural Practices [111] may help to limit or even reduce contamination and the dissemination of antibiotics and resistance determinants through the human–animal–environment chain.

## 6. Conclusions

Manure application has been shown to increase the diversity and abundance of ARGs and MGEs in manure-amended soils. Although ARG persistence in soil may vary from a few days to some months, they can be taken up by bacteria through HGT mechanisms, including soil microbiota and potential animal and human pathogenic bacteria, which may reach humans via the food chain. Different organic fertilizers impact ARG diversity and HGT events differently and this point should be taken into account when choosing a fertilizer. Different composting techniques have been shown to lead to varied reduction levels of specific ARGs, highlighting the importance of knowing local antimicrobial resistance patterns before choosing which composting technique to use. Furthermore, more studies on composting parameters are needed to improve its efficiency.
